# The Optical Characterization and Distribution of Dissolved Organic Matter in Water Regimes of Qilian Mountains Watershed

**DOI:** 10.3390/ijerph19010059

**Published:** 2021-12-22

**Authors:** Min Xiao, Zhaochuan Chen, Yuan Zhang, Yanan Wen, Lihai Shang, Jun Zhong

**Affiliations:** 1 Tianjin Key Laboratory of Water Resources and Environment, Tianjin Normal University, Tianjin 300387, China; chenzhaochuan@tjnu.edu.cn (Z.C.); wenyanan@tjnu.edu.cn (Y.W.); 2 Chongqing Municipal & Environmental Sanitation Monitoring Department, Chongqing 401121, China; cqjsfwk@126.com; 3 State Key Laboratory of Environmental Geochemistry, Institute of Geochemistry, Chinese Academy of Sciences, Guiyang 550081, China; shanglihai@vip.skleg.cn; 4 Institute of Surface-Earth System Science, School of Earth System Science, Tianjin University, Tianjin 300072, China; jun.zhong@tju.edu.cn

**Keywords:** dissolved organic matter, Qilian Mountains watershed, spectroscopic technique, autochthonous generation, mineral disintegration

## Abstract

The constituents and content of dissolved organic matter (DOM) in the Qilian Mountain watershed were characterized with a spectroscopic technique, especially 3-DEEM fluorescence assisted by parallel factor (PARAFAC) analysis. The level of DOM in the surrounding area of Qinghai lake (thereafter the lake in this article specifically refers to Qinghai Lake)was highest at 9.45 mg C·L^−1^ and about 3 times less (3.09 mg C·L^−1^) in a cropland aquatic regime (the lowest value). In general, DOM was freshly autochthonously generated by plankton and plant debris, microorganisms and diagenetic effects in the aquatic environment (FI > 1.8). Component 1 (humic acid-like) and 3 (fulvic acid-like) determined the humification degree of chromophoric dissolved organic matter (CDOM). The spatial variation of sulfate and nitrate in the surrounding water regime of the lake revealed that organic molecules were mainly influenced by bacterial mediation. Mineral disintegration was an important and necessary process for fluorescent fraction formation in the cropland water regime. Exceptionally, organic moiety in the unused land area was affected by anespecially aridclimate in addition to microbial metabolic experience. Salinity became the critical factor determining the distribution of DOM, and the total normalized fluorescent intensity and CDOM level were lower in low-salinity circumstances (0.2–0.5 g·L^−1^) with 32.06 QSU and 1.38 m^−1^ in the grassland area, and higher salinity (0.6~0.8 g·L^−1^) resulted in abnormally high fluorescence of 150.62 QSU and absorption of 7.83 m^−1^ in the cropland water regime. Climatic conditions and microbial reactivity controlled by salinity were found to induce the above results. Our findings demonstrated that autochthonous inputs regulated DOM dynamics in the Qilian Mountains watershed of high altitude.

## 1. Introduction

Fresh water is an essential resource for modern civilization. The human intervention scarcity of water was driven by land use land cover change, as well as population increases [1]. Global water scarcity is driven by water quality issues (water temperature, salinity, organic pollution and nutrients), and polluted return flows degrade water quality and exacerbate water scarcity. Technical and economic constraints of expanding desalination and treated wastewater reuse across the world still exist and water purification treatment is confronted with more challenges [2]. Natural organic matter (NOM, which includes DOM and particulate organic matter) is a key material in sustaining all biogeochemical processes and phenomena in ecosystems [3]. NOM originates from two primary sources, i.e., plant materials in terrestrial ecosystems and plankton in aquatic systems [4]. Dissolved organic matter (DOM) is the largest pool of organic matter in the sea and it plays an important role in carbon biogeochemical cycles of aquatic environments. DOM is composed of a series of organic compounds resulting from the release of bacteria, phytoplankton and macrophytes and their continuous transformation through photochemical and microbial processes [5]. Chromophoric dissolved organic matter (CDOM) is the primary DOM in aquatic environments, and it is one of the major determinants of optical descriptors of natural waters and can affect the availability and spectral quality of DOM in the water [6]. DOM is a diverse mixture of compounds forming gradients of composition, structure and biogeochemical reactivity derived from allochthonous, autochthonous and microbial sources [7]. Thus, probing biogeochemical processes of NOM are critical for water quality evolution, and water quality monitoring and analysis are the crucial point between DOM and land use in watersheds.

Optical measurements (absorbance and fluorescence) have been extensively used for characterizing DOM due to rapid sample throughput, low analytical cost and ability to trace DOM composition. The operationally defined humic and fulvic acids have long been credited as a major source of red-shifted fluorescence from allochthonous sources. Excitation–emission matrix (EEM) fluorescence is obtained by successive emission spectra at a series of excitation wavelengths and widely is used to characterize CDOM in aquatic environments [6,8]. In the previous research, microbial-like byproducts from increased microbiological activity formed during nitrification, whereas the terrestrial humic-like compounds predominated in the non-nitrified water samples [9].

To date, reports on the dynamics of individual fluorophores in the water of the region of the Qilian Mountains that have been identified by PARAFAC are limited. Accordingly, this study aimed to (1) apply the PARAFAC model to characterize the fluorescent constituents and properties of CDOM in water samples; (2) study the source and distribution of identified fluorescent components and (3) further cognize the process and factors that control the origins of CDOM and decipher the pressure on water quality from land use pattern change. 

## 2. Materials and Methods

### 2.1. Description of the Study Sites

**Figure 1:**
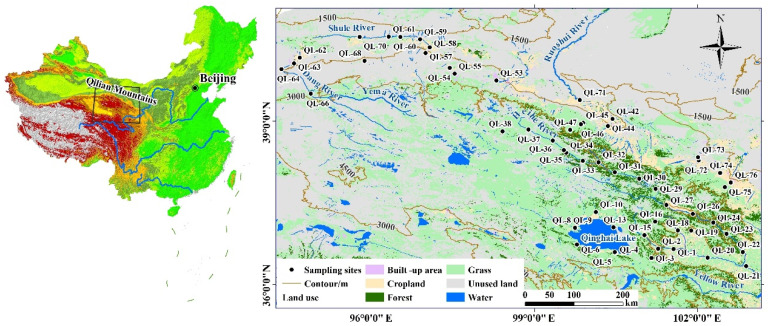
Map of the study watershed and the sampling locations.

The Qilian Mountains (36–40° N, 93–104° E), located on the border between Qinghai Province in the northeast and Gansu Province in the west, are one of the major mountain ranges in China. The range is composed of series of parallel mountains and valleys with a northwest trend in the northeastern margin of the Tibetan Plateau [10]. It is 800 km long from east to west and 200–400 km wide from north to south. It is 4000–6000 m above sea level and covers an area of 2062 square kilometers, and the altitude range for the sampling activities was 1170–3780 m. The Qilian Mountains are the transition zone from the Loess Plateau to the Mongolian–Xinjiang Plateau and the Qinghai–Tibet Plateau, belonging to an important part of the Qinghai–Tibet Plateau. The climate of this river basin is dry and has little rainfall, attributed to the interaction of the Siberian high and East Asia monsoon in the northwest part, whereas the warm moist flow from the southeast provides abundant rainfall and moisture [11]. The annual precipitation ranges from 2.27–74.41 mm in the watershed, with a maximum of 110 mm occurring in the east part concentrated from July to August [12]. The Qilian Mountains are so densely covered with rivers and wetlands that they deserve the title of “China’s Wet Island”, and they nourish the thirsty western land with their special ecological status and climatic conditions [13] (Figure 1). The Qilian Mountains are rich in glacial resources, under the action of glaciers, snow melt water and precipitation, and large areas of forests, grasslands and wetlands have been formed in the Qilian Mountains, which play a role in water conservation [14]. The eco-environment has not been protected well around the Qilian Mountains through the ages, e.g., overstocking, severe riverbed damage, Heihe River cutoff and even drying up. 

### 2.2. Analytical Studies

Water samples were collected from the Qilian Mountain region and around Qinghai Lake, which covers an area from 36.3° N to 40.6° N and from 94.3° E to 102.9° E (Figure 1). A total of 55 stations (*n* = 55) were randomly selected to sample in the snow season, 2019. 

High-density polypropylene bottles were used to collect samples using the standard method (APHA-AWWA-WEF, 1998). For hydro-chemical analysis, 0.45 µm membrane filters (Millipore, Boston, MA, USA) were used for filtering samples to characterize DOM. The samples were kept in the lab using cooler boxes and preserved in a freezer for analysis. The physical and chemical parameters of wetland water samples, including temperature (T), electrical conductivity (EC), pH, oxidation and reduction potential (Eh), dissolved oxygen (DO) and salinity (SAL), were measured in the field using portable pH/EC/DO meters (WTW 3430, Munich, Germany). Further laboratory analysis items included dissolved organic carbon (DOC) concentration, UV absorption and fluorescence. All the portable meters were calibrated using appropriate standards. Cl^−^, NO_3_^−^ and SO_4_^2−^ were measured by ion chromatography (Dionex ICS-90 system, Thermo Scientific Company, Waltham, MA, USA) equipped with a 4 mm ASRS-ultra II suppressor and a DS-5 conductivity detector. Triplicate analysis was carried out on samples for selected spectrum to provide accuracy and precision of investigation and represented data were the average results. Water quality parameters are ordinarily used to explain water status and expound the biogeochemical processes on organic matter. Sample excitation–emission matrix (EEM) spectroscopy fluorescence spectra were made using a fluorescence spectrophotometer (F-7000, Hitachi, Japan) with the help of FL solution software. The PARAFAC modeling was investigated employing the N-way toolbox of MATLAB version 3.1. The data EEMs of the tested samples were made with excitation wavelengths varying from 220–400 nm every 5 nm and emission wavelengths ranging from 280–500 every 1 nm in this research. The sampling datasets were run in the PARAFAC model in three consecutive phases. The variability elucidated by the PARAFAC analysis was 90.3% for water samples from the Qilian Mountains. 

### 2.3. Absorbance and Fluorescence Spectroscopy

The water samples were allowed to reach room temperature and the instrument was switched on for 30 min before the sample analysis. Absorption spectra were obtained between 190 and 700 nm at 0.5 nm intervals using a Persee Analytics T9cs double-beam UV-Vis spectrophotometer equipped with a 1 cm path length quartz cuvette (volume of 4 mL), and Milli-Q water was used as the blank. The data were corrected for scattering and baseline fluctuations by subtracting the absorption coefficients at 700 nm. The absorption coefficients (a) were calculated from the absorbances (A) obtained from the spectrophotometer using:

a(λ) = 2.303 × A(λ)/*l*

where A(λ) is the absorbance at the wavelength λ, and *l* is the path length in meters. 

For carbon-specific UV absorbance at 254 nm (SUVA_254_), the absorption coefficient at 355 nm was calculated as an index for the abundance of CDOM and the spectral slope over 275–295 nm (S_275–295_) was calculated as an index for the average molecular weight of organic matter [15]. A shoulder peak at 280 nm in the absorption spectra of CDOM was mainly associated with primary production and algal debris [16]. Thus, Δa_280_ was calculated to assess the intensity of the shoulder peak [17]. Briefly, the absorbance over 300–400 nm was fitted with the exponential function (R^2^ = 0.882–0.999), which was extrapolated to 280 nm to obtain the fitted value of a_280_. Δa_280_ was the difference between the measured and the fitted values of a_280_. Excitation–emission matrices (EEM_S_) were collected using an F7000 fluorospectrophotometer. Scans were collected over 5 nm increments with excitation wavelengths from 200–400 nm and emission wavelengths from 280–500 nm. The fluorescence spectrum of each sample was corrected for inner-filter effects based on the absorption spectrum, the blank signals of Milli-Q water were subtracted and it was normalized by the fluorescence intensity of 1 µg/L quinine sulfate. Spectral indices, such biological index (BIX), fluorescence index (FI) and humification index (HIX), and parallel factor analysis (PARAFAC), were used to analyze the optical data. SUVA_254_ and HIX reflect aromaticity and unsaturation, as well as the humic character or autochthonous DOM component [18], and higher values often indicate terrestrial sources. Conversely, high BIX and FI are often related to higher microbially produced DOM. PARAFAC analysis was conducted using the drEEM toolbox in MATLAB and decomposed the signal of three fluorescence components. The three-component model explained 90.30% of the variance, and we did not consider a four-component model based on visual inspection of the fourth component because the four-component model had a low core consistency (44.8%). The percent contribution of each component to the sum of the *F*_max_ values (maximum fluorescent intensity of each component) was calculated for each component and is reported as component C1–C3 (Table 1). Basic optical indexes (HIX, BIX, SUVA, FI, a355) are used to characterize the organic nature. PARAFAC analysis should be noted as a difficulty of this study. sources, components and distribution, as well as drop degree of water quality influenced by human intervention, are reflected by fluorescence analysis of DOM assisted by analysis of hydrochemical and UV optical parameters.

### 2.4. Data Management and Statistical Analysis

All data generated or analyzed during this study are included in the main text. PARAFAC modeling, calculation of optical indices and fluorescent plots were conducted in MATLAB, using the drEEM toolbox. Correlation analysis, a *t*-test and principal component analysis (PCA) were performed using the spectroscopic indices by IBM SPSS 23. PCA factor-number appropriateness was determined by the eigenvalue (>1) and the captured variance (>75%). Correlations were examined using Pearson correlation coefficients to assess the relationships between the physicochemical parameters and spectroscopic indices. The relative abundances of PARAFAC components and spectroscopic indices were compared using a one-way ANOVA for all water samples.

## 3. Results

### 3.1. Hydro-Chemical Characteristics of the River and Lake Water Samples

#### 3.1.1. Inorganic Parameters

Fifty-five water samples were collected from water regimes of forest/grassland, lake surrounding area, cropland and unused land, as illustrated in Figure 1. The sampling period was the snow season, in mid- to late October of 2019. The pH of the water is slightly acidic to alkaline, ranging from 6.24 to 8.90 with an average value of 8.59, indicating that the water of the study region is weakly alkaline. The concentrations of EC ranged from 0.31 to 1126 µS·cm^−1^ with a mean value of 539.73 µS·cm^−1^. The oxidation reduction potential (ORP) ranged from −166.00 to 85.18 mV, detected at station 22 and 55, respectively, reflecting the huge difference in the oxidation–reduction property in water samples scattered across lands of different types of utilization. As illustrated in Figure 1, station 22 and 55 are located at a water regime in grassland/forest land and unused land. The concentrations of Cl^−^, NO_3_^−^, SO_4_^2−^ ranged from 12.61~43.85, 4.39~13.99, 18.56~58.06 mg·L^−1^, and averaged 25.73 ± 14.35, 6.96 ± 4.03 and 33.56 ± 15.64 mg·L^−1^ in the lake surrounding area. They ranged from 4.76~85.33, 1.02~13.45, 19.43~250.6 mg·L^−1^, and averaged 20.33 ± 19.23, 5.98 ± 2.40, 115.19 ± 73.82 mg·L^−1^ in the water of the grassland area. In the cropland area, they ranged from 6.31~86.57, 3.08~22.57, 43.47~222.05 mg·L^−1^, and averaged 32.66 ± 26.28, 8.76 ± 5.59, 149.01 ± 61.32 mg·L^−1^. They ranged from 12.23~127.42, 3.06~7.99, 93.10~212.62 mg·L^−1^, and averaged 62.66 ± 35.26, 4.58 ± 1.75, 160.44 ± 49.36 mg·L^−1^ in the unused land area. The values fall within the range of basic levels of human health according to the Quality Standard for Groundwater GB/T 14848-93. Cl^−^ in the water regime of the grassland and cropland area was much lower than that in the unused land area (independent *t*-test, *p* < 0.05). NO_3_^−^ in the water of different land-use types did not show any significant discrepancies. SO_4_^2−^ in the lake surrounding area differed a lot from the levels of other land-use patterns (independent *t*-test, *p* < 0.05), but differed little for grassland, cropland and unused land areas. 

#### 3.1.2. Organic Parameters

DOC concentrations of water samples showed high spatial heterogeneity and varied from 2.53~17.89, 1.60~16.34, 0.95~8.40, 1.63~7.60 mgC·L^−1^ in water of the lake surrounding area, grassland, cropland and unused land watershed, respectively, with the mean values 9.45 ± 6.20 (mean and standard deviation, m ± std), 6.14 ± 3.95, 3.09 ± 2.23, 4.13 ± 2.49 mgC·L^−1^. The absorption coefficient a_355_ ranged from 0 to 7.83 m^−1^, with an average value of 1.46 ± 1.56 m^−1^. a_355_ was higher in the lake area without a striking difference compared to cropland (1.19 ± 2.00 m^−1^) and grassland (1.38 ± 1.15 m^−1^) areas (independent *t*-test, *p* > 0.05), but it was significantly higher than that of the unused land (0.72 ± 0.57 m^−1^) area (independent *t*-test, *p* < 0.05). 

**Figure 2:**
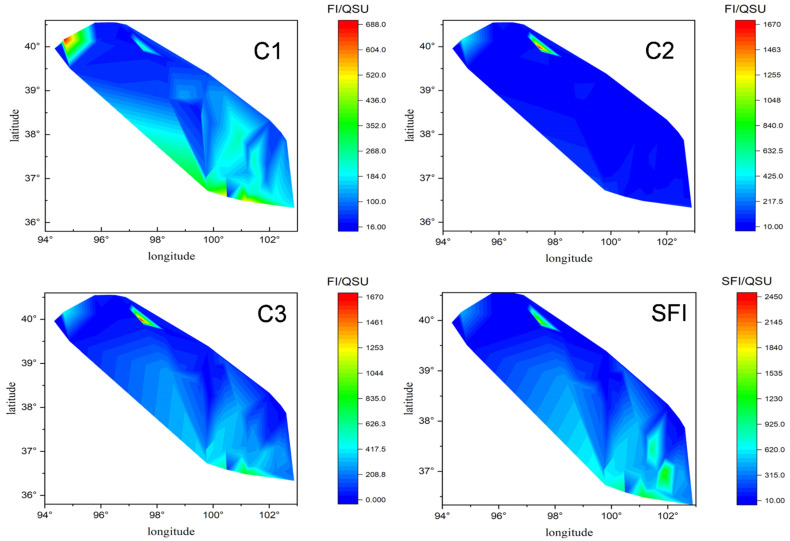
Spatial distribution of three fluorescent components along with the sampling transect (SFI: Sum of fluorescent intensity).

The spatial distribution of component 1, 2, 3 in this river basin showed notable spatial variations (Figure 2). Comprehensively, the *F*_max_ values of C1–C3 were 16.99~687.18, 12.68~1669.80, 0~1318.45 QSU, with average values of 142.61 ± 131.05, 74.23 ± 224.79, 286.31 ± 244.44 QSU, respectively. The fulvic-like component was dominant, and the intensity order is generally C3 > C1 > C2. The gross of the three components showed a similar trend to *F*_CDOM(355)_ along the transect, with four abnormal prominent values appearing at QL-3, 5, 54, 62 with a gross of *F*_max_ 1934.71, 1763.84, 2444.52, 1132.75 QSU, and 629.60~1941.37 QSU higher than the average (503.15 QSU) for all samples.

With an exception, the component C2 in the water of the unused land area was the highest and its fluorescence intensity averaged 265.96 ± 619.33 QSU, although compared to other types of land the difference is not significant (paired *t*-test, *p* > 0.05). The components C1 and C3 averaged 69.80 ± 91.73 and 142.69 ± 162.66 QSU in the unused land area. According to land-use types, the gross *F*_max_ of the three components ranked as the area surrounding Qinghai Lake > grassland > unused land > cropland with the concrete data of 802.89 ± 523.97, 505.42 ± 342.93, 478.45 ± 868.08, 361.10 ± 279.57 QSU. The gross *F*_max_ in the water around Qinghai Lake was much higher than that in cropland (independent *t*-test, *p* < 0.05), but not very significant when compared with the gross amount in grassland and unused land water regimes (independent *t*-test, *p* > 0.05) (Figure 2). Influx, along with river water of high mineral content (TDS = 932.96 mg·L^−1^ KCl) plus atmospheric deposition, resulted in the area around Qinghai Lake becoming the collection location of DOM. Generally, CDOM absorption (fluorescence at 355 nm) increased from a low of 0.72 ± 0.57 m^−1^ (15.30 ± 20.10 QSU) in unused land water regimes to a high of 3.02 ± 1.88 m^−1^ (50.85 ± 35.98 QSU) of the Qinghai Lake surrounding water regime, with mean values of 1.38 ± 1.15 m^−1^ (32.06 ± 21.58), and 1.19 ± 2.00 m^−1^ (27.84 ± 37.17 QSU) in the grassland area and cropland water regime fell in the middle range.

Water samples exhibited SUVA values (m ± std) of 2.64 ± 1.78, 1.98 ± 1.45, 2.64 ± 1.61, 1.13 ± 0.52 L·m^−1^·mg C^−1^ (Table 1). S_275–295_ values (m ± std) of 0.02 ± 0.01, 0.02, 0.02 ± 0.01, 0.02 nm^−1^ in water samples of the abovementioned four categories of land use were found, fluctuating at 0.02 nm^−1^. Throughout the sampling transect, the SUVA_254_ values ranged from 0.21 to 5.92 L·m^−1^·mg C^−1^, with an average value of 2.12 ± 1.51 L·m^−1^·mg C^−1^. The spectral slope S_275~295_ ranged from 0.01~0.048 nm^−1^, with an average value of 0.02 ± 0.006 nm^−1^. The S_275~295_ showed no significant difference for different land use types (independent *t*-test, *p* ≥ 0.05). However, in terms of SUVA_254_ values, it showed significant difference between cropland and unused land area (independent *t*-test, *p* < 0.05). 

A two-dimensional fluorescence index (FI) is also used to identify the sources of DOM, and was calculated as a ratio of emission fluorescence intensities at 450 to 500 nm, which were excited at 370 nm [19]. The mean FI value in this study is recorded at 1.92 with maximum and minimum values of 2.29 and 1.53, respectively.

The humification index (HIX) was calculated as the ratio of the fluorescence peak area over the emission wavelengths of 435–480 nm to those over 300–345 nm at the excitation wavelength of 255 nm [20]. In this study, the humification index ranged between 0.78 and 6.96, with an average value of 3.23 ± 1.73. Only twenty water samples (QL-1, QL-2, QL-3, QL-5, QL-9, QL-10, QL-15, QL-20, QL-22, QL-23, QL-24, QL-27, QL-29, QL-30, QL-31, QL-32, QL-33, QL-34, QL-46, QL-75) showed a humification index ranging from 4 to 7, situated in the forest and grassland area. More than half of the samples provided HIX values from 0.78 to 3.9. HIX values of the water around Qinghai Lake and in the grassland region averaged 4.28 ± 2.09 and 4.18 ± 1.50, respectively, which were significantly higher than that of the unused land of 1.41 ± 0.52 (independent samples *t*-test, *p* < 0.05), and higher than the value of 2.92 ± 1.47 for cropland, but without a significant difference (independent samples *t*-test, *p* > 0.05). 

The biological index (BI) was calculated by the ratio of fluorescent intensity at emission wavelength 380 nm to 430 nm and at excitation wavelength 310 nm [8]. In this study, the BIX values were within the range of 0.79–3.13 with a mean value of 0.99 ± 0.31. The highest BIX appeared in the unused land area, 1.35 ± 0.8, but there was no obvious difference between this type of land use and the lake surrounding area, cropland and grassland area (independent samples *t*-test, *p* ˃ 0.05). 

## 4. Discussion

### 4.1. Fluorescent Indices

An FI value less than 1.2 (FI < 1.2) indicates a terrestrial origin and high aromaticity of FDOM whereas FI > 1.8 corresponds to a microbial DOM source and lower aromaticity [19,21]. Nearly all of the examined water samples in this study have FI values above 1.8, which indicates the predominantly microbial-derived origin of FDOM and low aromaticity. This index expresses the shifting of fluorescing molecules to larger wavelengths as humification of dissolved organic matter continues, resulting from lower H/C of the emission spectra. High values of the humification index are an indication of increased humification [19]. The HIX for the humic content of DOM has low values (<4) for non-humified DOM of biological or aquatic bacterial origin, but high values (>10) for DOM with a strong humic character or with an important terrigenous contribution [22]. The results indicate a wide range of humification degree and mainly mixed sources of biological and allochthonous DOM, reflecting the presence of a labile fraction of low HIX. Higher FI values at the forest and grassland stations revealed that decomposed plants contributed much to humus. Lower FI values of cropland and unused land demonstrated a significant non-humic character of DOM with an important contribution from strong autochthonous sources and weak terrigenous components. BIX indicates the comparative influence of biologically produced dissolved organic matter. The BIX values range from 0.6 to 0.7 and 0.7 to 0.8 and are an indication of low and intermediate autochthonous components, respectively [23]. The results indicate strong autochthonous components in this study. HIX and BIX have been used to identify the source and fate of CDOM in estuarine water and enumerated changes in fluorescence characteristics of DOM [22]. Indices have been used to understand various ecosystem processes, including changes in DOM. 

### 4.2. CDOM Absorption and Fluorescence Variability in Different Water Regimes

**Figure 3:**
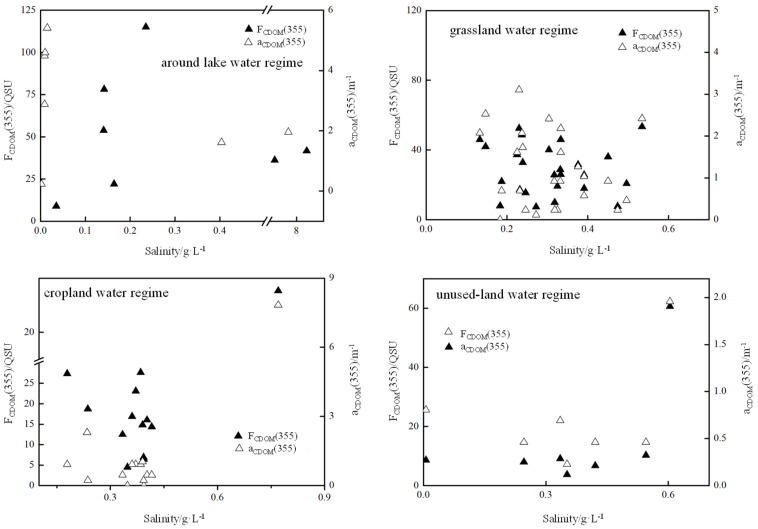
Variation of CDOM absorption and fluorescence with salinity along the axial transect. Empty triangles represent CDOM absorption, filled triangles represent CDOM fluorescence for different land use water regimes.

The absorption coefficient of CDOM generally decreased with increasing wavelength from 300–600 nm. Different regions of the Qilian Mountains usually show different concentrations of CDOM components. The CDOM and fluorescence at 355 nm (F_355_) in different water regimes are shown in Figure 3. The variation with salinity (Figure 3) showed similar increasing–decreasing fluctuation in the cropland and lake surrounding water regime. It was preliminarily inferred that the organic fraction was relevant with salinities of water [8,24]. Comparatively, a conservative distribution of CDOM optical descriptors (a_355_ of 1.38 m^−1^ and F_355_ of 32.06 QSU) occurred in grassland water regimes within the salinity range of 0.2–0.5 g·L^−1^, and higher salinity (0.6~0.8 g·L^−1^) resulted in abnormally high absorption and fluorescence in cropland (7.83 m^−1^ and 150.62 QSU) and unused land (1.96 m^−1^ and 60.64 QSU) regions. This result was consistent with those reported by Parlanti et al. (2000) [25], but contrary to a previous study that found decreased FDOM intensity with increasing salinity gradients. Salinity played important roles in shaping the microbial community at phylum and genus levels, as different species of bacteria favored discriminatory xenobiotic metabolism and signal transduction of high or low salinities, thus impacting the generation or degradation of DOM [26]. Once the salinity arrived at the highest value of around 8, the activation of organic materials attenuated, as illustrated in Figure 3, in the region around the lake. 

### 4.3. Component Variability along the Transect

The FDOM components in this investigation were characterized with three-dimensional excitation–emission matrix spectroscopy (3DEEM) coupled with the parallel factor analysis (PARAFAC) technique [27,28]. A total of 55 EEMs were modeled with PARAFAC using MATLAB R2016b with the DOMFluor toolbox 1.7. The reliability of the PARAFAC model was verified and the number of individual fluorescent components was determined by split-half validation and residual analysis [29], and the fluorescence intensity of each component was represented with *F*_max_ (QSU, quinine sulfate unit).

**Figure 4:**
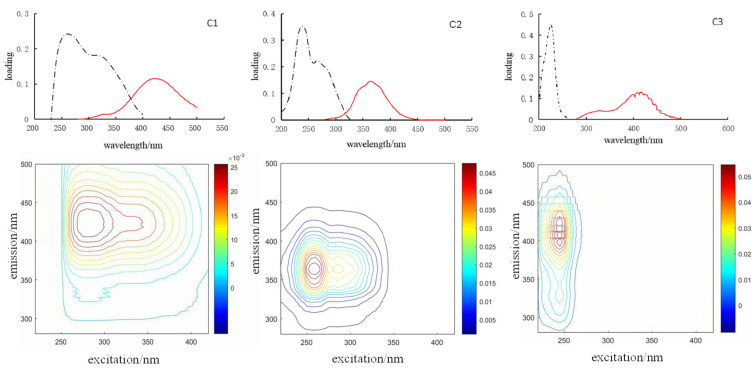
Excitation (black dotted lines), emission loadings (red solid lines) and contour plots of component 1, component 2 and component 3 for the whole dataset.

Table 2 shows the three components of EEM spectra that were identified by the PARAFAC model. The spectral characteristics of the component that were identified in this study were extremely similar to those characteristics of CDOM in other aquatic environments that were previously identified [6,27,28]. The excitation and emission pairs of the main peak positions for each component were summarized and compared with those pairs that were found in earlier studies, as shown in Table 2. Based on the fluorescence features, the components can be distinguished as one humic-acid (HA)-like component C1 and one protein-like component C2 as well as ultraviolet fulvic-acid (FA)-like C3. The three-dimensional fluorescence spectra of three kinds of fluorescent components and the maximum are shown in Figure 4. Although three individual components were identified for this dataset using the PARAFAC model, it does not suggest that only three types of fluorophores were present in these samples. C1 had a primary fluorescence peak at Ex/Em wavelengths of 265/425, 315/425 nm, which was similar to samples covering a wide range of the spectrum of DOM molecular composition, encompassing modern, highly aromatic allochthonous DOM, to aged DOM with low aromaticity, dominated by autochthonous production. Component 1 is defined as a combination of the classically described terrestrial and marine humic-like fluorescence at an excitation maximum of 265 nm (secondary at 315 nm) and an emission maximum at 425 nm [30], and the tryptophan-like peak at Ex/Em wavelengths 265/334 nm, namely, C1 originating from terrestrial inputs and autochthonous production (e.g., exudation of phytoplankton and biological contribution). C1 (ex: 230/300 nm, em: 418 nm) was reported to include a microbially derived humic-like peak, due to the contribution of authigenic humic-like matter [31,32]. C1 reflected the fluorescence properties of the long-wave humus, representing fluorescence components with the maximum excitation and emission wavelength. Component 2 includes two fluorescent peaks, the first group of Ex/Em wavelengths 240/363 nm, which is typical of protein fluorescence and similar to what is classically described as tryptophan-like fluorescence. Another peak 265/363 nm, also a kind of protein-like material, is representative of dissolved metabolic materials by microbes. C2 was a biodegradable protein-like substance, and was related to an aromatic ring amino acid structure in DOM, and the fluorescence peaks occurred in the region with a short emission wavelength. Ultraviolet fulvic acid-like C3 (ex: 225; em: 332; 408; 417; 423; 431) is assigned to autochthonously produced tyrosine (225/332 nm) and fulvic acid (225/408, 417, 423, 431 nm) materials with a single excitation wavelength. Stedmon et al. (2000) found it in sewage and agricultural wastewater, which contained a large number of phenolic hydroxyl, carbonyl and other functional groups [33]. C1 and C3 were humic-like humic acids and fumaric acids, related to hydroxyl and carboxyl groups in the structure of humus. 

### 4.4. Relationship between the Spectral Indices of DOM and Other Parameters

**Figure 5:**
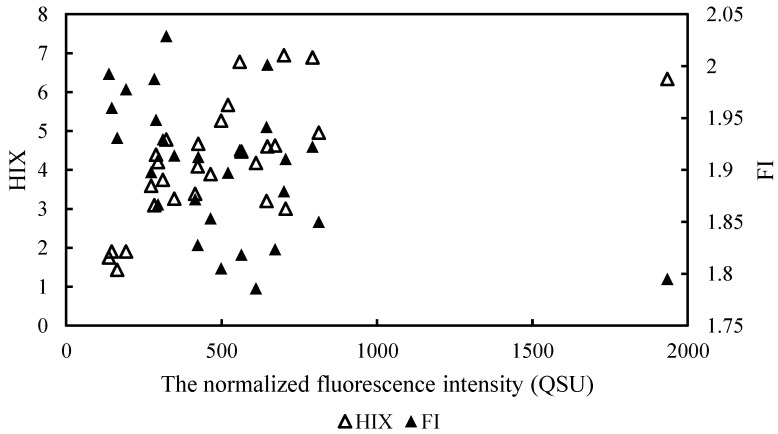
Humification indices and fluorescence indices versus the total normalized fluorescence intensity in grassland water regime.

Correlation analysis between the spectral indices of DOM and water quality parameters was carried out to infer the factors underlying the dynamics of DOM. a_355_ (CDOM) in the lake surrounding area did not show any correlations to fluorescent components (*p* > 0.05), while some strong correlations were found in other types of land use. For example, a_355_ correlated positively with C1 and C2 in cropland (r = 0.992, *p* < 0.01), and correlated positively with C1, C2 and C3 (r = 0.956, *p* < 0.01) in grassland and unused land (r = 0.965, *p* < 0.01). This implied that CDOM and C1–C3 had an overall similar spatial change and fluorescent moiety formation and consumption influenced the distribution trend of CDOM. Δa_280_, the index for the intensity of the shoulder peak in the CDOM absorption spectra, correlated strongly with C1 and C2 in cropland (r = 0.768, r = 0.67, *p* < 0.01), and with C1 in grassland (r = 0.39, *p* < 0.05). It did not show any correlations with fluorescent components in the lake surrounding area and unused land area. Total normalized fluorescence intensity, as suggested by Kowalczuk et al. (2009) [38], was found by summing fluorescence intensities of all the three identified components in each land-use type and is plotted against HIX an FI for the grassland water regime in Figure 5. A decrease in HIX as a function of total fluorescent intensity from southeast to northwest in grassland water regimes suggested the role of in situ plankton production at these locations and then FI values increased. The denser the grass/forest, the more endogenous it was. Otherwise, obvious correlations between HIX/FI and total normalized fluorescence intensity were not observed for water regimes in other land-use types.

**Figure 6:**
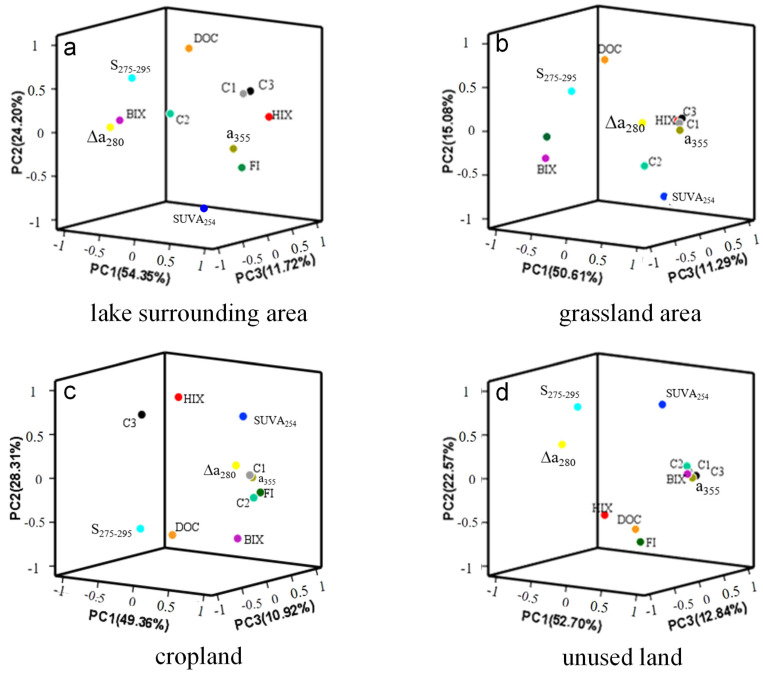
PC1 and PC2 loadings of DOM, and PC1 and PC2 scores for different samples. Positive and negative numbers on axes indicate the correlation coefficients which are positive or negative. (**a**): lake surrounding area; (**b**): grassland area; (**c**): cropland; (**d**): unused land.

Principal component analysis was performed using the *F*_max_ of C1–C3, HIX, BIX, a_355_, DOC, etc. Three principal factors were identified in the lake surrounding area, which explained 54.7% (PC1), 26.6% (PC2), 10.33% (PC3) of the total variance of the dataset, respectively (Figure 6a). PC1 correlated positively with HIX, C1 and C3 but negatively with C2 and DOC. Generally, microbial metabolic activities (zoo- and phytoplankton). such as decomposition and synthesis, assimilation and dissimilation, were strengthened with increasing FI values. However, a significant anti-correlation between BIX and FI was observed in the lake surrounding area (r = −0.898, *p* ≤ 0.01), which resulted from the intense bioactivities and strong terrestrial input at QL-4. The highest BIX of 1.21 and the lowest FI of 1.53 at this sampling station indicated that allochthonous DOM was bioavailable. PC1 represented the autochthonous organic matter which was biorecalcitrant and dominated by humic-like components. The plant debris and algal sources of DOM were reflected in the absorbing spectral parameter Δɑ_280_, and the autochthonous (Δɑ_280_, BIX) and allochthonous (HIX) indices showed strong negative correlations (r = −0.852, *p* ≤ 0.05). This was similar to that reported by Lee et al. (2018), that the degree of the shoulder peak over 240–290 nm for algal DOM decreased with an increasing proportion of allochthonous sources in the DOM samples mixed with two contrasting sources [39]. The positive correlation between BIX and S_275–295_ (r = 0.847, *p* ≤ 0.05) revealed that molecular weight decreased gradually with strengthening of microbial activities. Meanwhile, aromatic DOM showed a decreasing proportion in the DOM reservoir with elevated DOC content (SUVA_254_ and DOC, r = −0.809, *p* ≤ 0.05), probably due to the added part of DOM being dependent on microbial generation or photochemical processing which induced the CDOM composition to change [40]. C1 correlated most strongly with C3 (r = 0.987, *p* ≤ 0.01) in water samples surrounding the lake, and SO_4_^2−^ was reduced when particulate organic matter was oxidized to produce dissolved fluorophores (C1, C3 vs. SO_4_^2−^, r = −0.857, *p* ≤ 0.05; SO_4_^2−^ vs. DOC, r= −0.768, *p* ≤ 0.05) that resulted in a decrease in oxidation reduction potential (ORP) (SO_4_^2−^ vs. ORP, r = 0.895, *p* ≤ 0.05), indicating sulfate-reducing bacteria mediated much during this process. This phenomenon was accordant with reports in groundwater thatSO_4_^2−^ in this aquatic environment suggested a positive correlation with HA, FA and degraded FA [8]. There is a sign that a protein-like substance was influenced by nitrate-reducing bacteria, as nitrate was reduced when bioavailable C2 was oxidized into inorganic carbon (NO_3_^−^ vs. C2, r = 0.915, *p* ≤ 0.05). Nitrate-reducing bacteria probably directly assimilated C2 (carbon substrate) which promoted the microbial reactivity of dissimilatory reduction on NO_3_^−^.

Three principal factors were identified which explained 50.61% (PC1), 15.08% (PC2) and 11.29% (PC3) of the total variance of the dataset in the water of grassland, respectively (Figure 6b). PC1 significantly and negatively correlated with DOC, indicating that DOM content was not the main factor for controlling the aromaticity and humification degree of DOM. Molecular size and aromaticity were observed to inversely correlate with HIX vs. S_275–295_ and HIX vs. SUVA_254_ (r = −0.58, *p* ≤ 0.01), clarifying that molecular weight increased and the number of double bonds was reduced with the deepening of humification for DOM in the grassland area. Similar to the circumstance of the lake surrounding area, the increasing trend of DOC instead resulted in the weakening of aromaticity (SUVA_254_ and DOC, r = −0.546, *p* ≤ 0.01). The increased portion of DOM may have been mainly lower aromatic content. HIX and BIX in the grassland area negatively correlated mutually (r = −0.527, *p* ≤ 0.01), and the molecular weight of DOM increased with higher HIX (HIX and S_275–295_, r = −0.483, *p* ≤ 0.01). The horizontal distribution trend of CDOM was influenced greatly by C1 and C3, and these two components (humic-like and fulvic-like) of CDOM became the determinants for humification degree of DOM (CDOM or C1/C3 and HIX, r = 0.651, *p* ≤ 0.01). C1, C2, C3 showed significant positive correlations mutually (r = 0.98, *p* ≤ 0.01) in the grassland area. C1, C2, C3 scores significantly anti-correlated with pH and DO (r = −0.797, *p* ≤ 0.01) in grass/forested land, suggesting that DOM was related to synthesis and mineralization of particulate organic matter (POM), namely, the primary productivity. Undoubtedly, stronger biological behavior resulted in higher FI values. The recalcitrant CDOM, including humic-like C1 and fulvic-like C3, inhibited the microbial behavior (Figure 6b) (C1 or C3 vs. BIX, r = −0.673, *p* ≤ 0.01), C1 and C3 were dominant in CDOM by absolute contents of 146.26 and 322.97 QSU over C2 with a value of 36.18 QSU. Plant debris or algal contributions predominated in the grassland water regime (Δɑ_280_ vs. a_355_, r = 0.463, *p* ≤ 0.05).

Different from the abovementioned two types of land use, HIX values in the water of the cropland and unused land area were generally less than 4, whereas BIX values at the two sampling areas were more than 1. This result indicated a remarkable autochthonous source of DOM which was freshly produced from metabolic activities of plankton and plant debris degradation. Likewise, three principal factors were identified, which explained 49.36% (PC1), 28.31% (PC2) and 10.92% (PC3) of the total variance of the dataset in the cropland area, respectively. Specifically, C1 correlated significantly with C2 (r = 0.953, *p* ≤0.01) in the water of the cropland area. Both were correlated to EC and salinity (r = 0.856, *p* ≤ 0.01), which indicated that the fluorescent components were released from the mineral substrate of aquifers with extended residence time of water [18]. Negative oxidation reduction potential (ORP) was concerned with oxygen consumption, and S was one of the mineral elements that was oxidized and liberated as SO_4_^2−^ into dissolution phase. SO_4_^2−^ multiplied gradually and contributed much to increasing EC and salinity (SO_4_^2−^ vs. EC, r = 0.869, *p* ≤ 0.01; SO_4_^2−^ vs. ORP, r = −0.621, *p* ≤ 0.05). The observed positive relationship between BIX and EC indicated that microbial behavior stimulated minerals to be liberated or disintegrate in the cropland area, e.g., extracellular polymeric substances encapsulated in particulates which promoted mineral disintegration, ascribed to the diagenetic effects of aquatic organism metabolism [18,41]. As for as PC1, HIX and DOC were the primary negative contributors. DOC and BIX were the significantly negative contributors for PC2 (Figure 6c). A higher molecular weight of DOM constituted the major factor for PC2. This result indicated that DOM was characteristic of the weak humification and autochthonous source of DOM by the active biological behaviors. Fluorescent component 3 in cropland had a more obvious characteristic of humification relative to other organic moieties (HIX and C3, r = 0.819, *p* ≤ 0.01). The significant correlation between a_355_ and Δa_280_ further expounded the rich endogenic DOM of a phytoplankton and algal source (a_355_ and Δa_280_, r = 0.729, *p* ≤ 0.01), and the fluorescent components C1 and C2 of CDOM behaved the same (C1 and Δa_280_, r = 0.768, *p* ≤ 0.01). Protein-like substance C2 was not limited in associating with aquatic vegetation [30], and was undoubtedly also derived from microbial excretion, degradation/synthesis and assimilation/dissimilation, etc. metabolic activities (C2 and BIX, r = 0.742, *p* ≤ 0.01). When the organic molecules aggregated into large ones, humification was strengthened and aromaticity increased (HIX and SUVA_254_, r = 0.601, *p* ≤ 0.05; SUVA_254_ and S_275–295_, r = −0.676, *p* ≤ 0.01), and the bioavailability of DOM was attenuated (HIX and BIX, r = −0.574, *p* ≤ 0.05) during the metabolic processes by microbes (FI > 1.9, microbial/phytoplankton metabolism predominated in the various transformations of DOM). Rich freshly produced autochthonous DOM primarily resulted from or in microbial/phytoplankton metabolism greatly (BIX and FI, r = 0.736, *p* ≤ 0.01), which could be ascribed to the combined diagenetic effects of aquatic organism metabolism and sunlight degradation [42].

Unused land was relatively smaller area, and CDOM and its fluorescent components (C1-C3) were predominantly recently produced by biological activities (BIX and CDOM, r = 0.983, *p* ≤ 0.01). Three principal factors were identified, which explained 52.70% (PC1), 22.57% (PC2) and 12.84% (PC3) of the total variance of the dataset, respectively (Figure 6d). PC2 correlated negatively with FI, indicating that stronger microbial metabolic behavior resulted in quota shrinkage of PC2. At the same time, the recently produced richer autochthonous DOM could create a larger size of organic molecules (FI and S_275–295_, r = −0.791, *p* ≤ 0.05). It was inferred that DOM of low humification (HIX averaged at 1.41) mainly originated from the microbial metabolic process of autogenous materials, and the common plants found in wetlands are a source of CDOM when they decompose [30]. Fluorescent components (C1, C2, C3) in the water regime of the unused land region displayed consistent variation along the axal transect (r = 0.99, *p* ≤ 0.01), and intimately correlated with Cl^−^ (r = 0.831, *p* ≤ 0.05), which was attributed to the dry climate in the northwest part where the water table declined and gradients narrowed, as described by Kabir et al. (2021) [8]. FDOM increased with increasing salinity constituents and long residence times. This proposed mechanism was supported by the increasing concentrations of Cl^−^ and DOC in Swan Coastal Plain wetlands [43]. Corresponding to the abovementioned contents, salinity was another regulator constraining microbial reactivity and FDOM distribution [26].

## 5. Conclusions

In this work, the occurrence of different types of autochthonous FDOM, that can be freshly produced by phytoplankton and microbial metabolic behavior, in river and lake waters has been ascertained. DOM was the richest in the lake surrounding area, as well as CDOM. Fluorescent materials were divided into three components, humic acid-like, protein-like and fulvic acid-like. DOM originated from plankton, macrophyte, etc. autochthonous freshly produced materials according to FI and BIX values. Δa_280_ further proved that DOM in the lake surrounding area, cropland and grassland was associated with phytoplankton and algal sources. In the grassland area, total normalized fluorescent intensity was evidently characteristic of decreasing humification and increasing authigenic production from phytoplankton, macrophytes and microorganisms. High molecular weight of DOM that was humified with low aromaticity was ascribed to microbial intermediation or photochemical processing. Formation and consumption of three components in the water regime surrounding the lake were mediated by sulfate—(C1 and C2) and nitrate-reducing bacteria (C3). Fluorescent components in cropland were derived from mineral dissolution by bacterial interruption. The large size of molecules was produced along with features of high humification and aromaticity. Atmospheric transportation and glacial melt in the unused land water regime created the platform for organic molecules. 

## Figures and Tables

**Table 1:** Indices of fluorescence and UV detection in water regimes of different land-use types.

	Lake Surrounding Water	Cropland Water	Grassland Water	Unused Land Water
C1 (QSU)	231.99 ± 164.18	127.03 ± 169.59	146.26 ± 98.45	69.80 ± 91.73
C2 (QSU)	55.99 ± 32.96	63.58 ± 110.95	36.18 ± 18.66	265.96 ± 619.33
C3(QSU)	514.91 ± 345.80	170.48 ± 123.61	322.97 ± 233.78	142.69 ± 162.66
a_355_ (m^−1^)	3.02 ± 1.88	1.19 ± 2.00	1.38 ± 1.15	0.72 ± 0.57
S_275–295_ (nm^−1^)	0.02 ± 0.01	0.02 ± 0.01	0.02	0.02
HIX	4.28 ± 2.09	2.92 ± 1.47	4.18 ± 1.50	1.41 ± 0.52
BIX	0.93 ± 0.13	1.00 ± 0.1	0.91 ± 0.06	1.35 ± 0.80

QSU: Quinine sulfate unit, 1 μg·L^−1^ quinine sulfate is used as 1 QSU. a_355_ is the absorbance coefficients at 355 nm. S275–295 denotes the absorption slope from 275–295 nm. HIX is the humification index. BIX is the biological index.

**Table 2:** Characteristics of the three fluorescence components from the water regimes of the Qilianshan region.

Components	Ex/Em	Description	References
Component 1 Humic acid-like	265/334; 275/425; 315/425	Terrestrial humic-like material Marine humic matter	C3: 310/380 [28]; C2: 265/475, C4: 275(355)/450 [34,35]; C3: <250/434 [18]; C1: 322/407 [36]; M: 310–320/380–420 [30]
Component 2 Protein	240/363; 265/363	Tryptophan-like substances free or bound in proteins Tyrosine-like materials	C5: 240/368 [27]; C8: 275/360 [28]; C3: ≤230(285)/340, C5: 275(≤230)/305 [37]; C4: 274/340 [18]
Component 3 Fulvic acid-like	225/332; 225/408; 225/417; 225/423; 225/431	Microbial humic-like substances	C1: ≤230(300)/418 [34,35]

## Data Availability

Data are available in a publicly accessible repository.

## Abbreviations

The following abbreviations are used in this manuscript:

DOM	Dissolved Organic Matter
CDOM	Chromophoric Dissolved Organic Matter
PARAFAC	Parallel Factor
EEMS	Excitation–Emission Matrix Spectroscopy
HL	Humic Acid-Like
FL	Fulvic Acid-Like
EC	Electrical Conductivity
Eh	Oxidation and Reduction Potential (ORP)
DO	Dissolved Oxygen
SAL	Salinity
